# *De Novo* Reconstruction of Consensus Master Genomes of Plant RNA and DNA Viruses from siRNAs

**DOI:** 10.1371/journal.pone.0088513

**Published:** 2014-02-11

**Authors:** Jonathan Seguin, Rajendran Rajeswaran, Nachelli Malpica-López, Robert R. Martin, Kristin Kasschau, Valerian V. Dolja, Patricia Otten, Laurent Farinelli, Mikhail M. Pooggin

**Affiliations:** 1 University of Basel, Department of Environmental Sciences, Institute of Botany, Basel, Switzerland; 2 United States Department of Agriculture–Agricultural Research Service, Horticultural Crops Research Laboratory, Corvallis, Oregon, United States of America; 3 Oregon State University, Department of Botany and Plant Pathology, Center for Genome Research and Biocomputing, Corvallis, Oregon, United States of America; 4 Fasteris SA, Plan-les-Ouates, Geneva, Switzerland; Washington State University, United States of America

## Abstract

Virus-infected plants accumulate abundant, 21–24 nucleotide viral siRNAs which are generated by the evolutionary conserved RNA interference (RNAi) machinery that regulates gene expression and defends against invasive nucleic acids. Here we show that, similar to RNA viruses, the entire genome sequences of DNA viruses are densely covered with siRNAs in both sense and antisense orientations. This implies pervasive transcription of both coding and non-coding viral DNA in the nucleus, which generates double-stranded RNA precursors of viral siRNAs. Consistent with our finding and hypothesis, we demonstrate that the complete genomes of DNA viruses from *Caulimoviridae* and *Geminiviridae* families can be reconstructed by deep sequencing and *de novo* assembly of viral siRNAs using bioinformatics tools. Furthermore, we prove that this ‘siRNA omics’ approach can be used for reliable identification of the consensus master genome and its microvariants in viral quasispecies. Finally, we utilized this approach to reconstruct an emerging DNA virus and two viroids associated with economically-important red blotch disease of grapevine, and to rapidly generate a biologically-active clone representing the wild type master genome of *Oilseed rape mosaic virus*. Our findings show that deep siRNA sequencing allows for *de novo* reconstruction of any DNA or RNA virus genome and its microvariants, making it suitable for universal characterization of evolving viral quasispecies as well as for studying the mechanisms of siRNA biogenesis and RNAi-based antiviral defense.

## Introduction

Owing to error-prone replication, viruses accumulate microvariants which deviate from a consensus master genome by one or more SNPs (single-nucleotide polymorphisms) and/or indels (insertions/deletions) and comprise a viral quasispecies that can rapidly evolve in changing environment [1]. Resistance-breaking strains often emerge from such microvariants and from recombination events involving distinct viral strains or viruses. Existing methods of viral diagnostics using antibodies and PCR often fail to identify new pathogenic strains and are not applicable for emerging viruses with unknown genomes. Therefore, next generation deep sequencing approaches and *de novo* assembly of virus genomes from sequencing reads hold a great promise for universal diagnostics of viral pathogens and reliable characterization of causative agent(s) of any given disease [2], [3]. In a pioneering work, Kreuze *et al*. [2] have demonstrated that a complete genome of a known plant RNA virus can be reconstructed *de novo* from multiple contigs of short interfering RNAs (siRNAs) which are generated in infected plants by the evolutionarily conserved RNA silencing/RNA interference (RNAi) machinery [4]–[6]. This and the follow-up studies have proven that deep siRNA sequencing and bioinformatics are applicable for identification and at least partial genome reconstruction of plant viruses and viroids [7]–[14] as well as insect viruses [15], [16]. Here we extend these findings by demonstrating that the complete genomes of plant DNA viruses of two major families – *Caulimoviridae* and *Geminiviridae* – can be reconstructed without a reference genome as a single contig or a few overlapping contigs of viral siRNAs. Furthermore, we show that bioinformatics analysis of viral siRNA population allows for the identification of the master genome and its microvariants in viral quasispecies. We also used this technology to reconstruct a newly emerged single-stranded DNA virus and two viroids associated with the red blotch disease of grapevines in the United States. Thus, deep siRNA sequencing can be used for identification and reconstruction of the consensus master genome of any plant virus or viroid, and for studying virus diversity and evolution. Moreover, our analysis of siRNAs derived from DNA viruses and viroids contributes to further understanding the mechanisms of siRNA biogenesis and RNAi-based antiviral defense and raises new questions for future research.

## Results and Discussion

**Figure 1:**
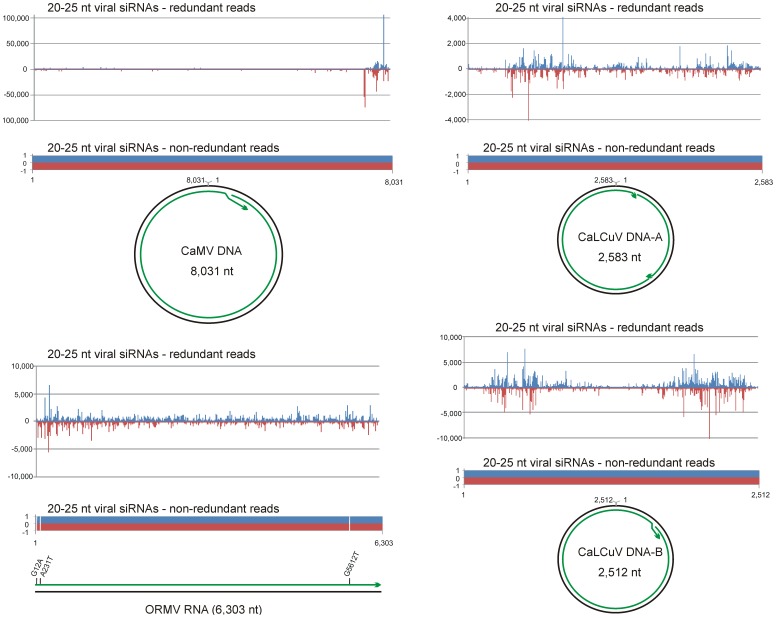
Maps of viral siRNAs and their contigs. The graphs plot the number of 20–25 nt viral siRNA reads (redundant and non-redundant) at each nucleotide position of the genomes of CaMV, ORMV and CaLCuV (DNA-A and DNA-B); Bars above the axis represent sense reads starting at respective positions; those below the antisense reads ending at respective positions. Circular DNA genomes of CaMV and CaLCuV and linear RNA genome of ORMV are shown below the graphs, with the siRNA contigs covering the genomes depicted as green lines with arrowheads. Mismatches between the ORMV contig and the reference genome are indicated.

Growing evidence indicates that 21–24 nt virus-derived siRNAs are produced from double-stranded (ds) RNA precursors covering the entire viral genome sequences [4], [17], [18]. Accordingly, complete or near complete genomes of new RNA virus strains have been reconstructed using a reference strain sequence as a scaffold for assembly of overlapping siRNA contigs which were generated by the short sequence read assembler Velvet [2], [7], [8], [10], [11]–[13]. Similar approaches have succeeded in detection and partial genome reconstruction of plant DNA viruses [2], [9], [10], [19]. Because DNA viruses do not replicate through dsRNA intermediates, viral siRNA precursors are likely produced by the host Polymerase II-mediated, bidirectional transcription of a circular viral DNA beyond the poly(A) sites, thereby including both coding (mRNA) and non-coding (promoter) sequences [4], [17], [18]. To validate this hypothesis and test if complete genomes of DNA viruses could also be reconstructed as single contigs of viral siRNAs we used the small RNA (sRNA) deep-sequencing libraries obtained from *Arabidopsis* plants infected with *Cauliflower mosaic virus* (CaMV) [18] and *Cabbage leaf curl virus* (CaLCuV) [17] (Datasets S1*A* and S1*B*), which represent the *Caulimoviridae* and the *Geminiviridae* families, respectively. Bioinformatic analysis of the redundant sRNA reads revealed that the hotspots of viral siRNA production cover only portions of the viral genome in both cases. The non-redundant reads, however, cover the entire circular genomes of CaMV and CaLCuV in the sense and antisense orientations without gaps (Figure 1; Dataset S2), albeit the density of non-redundant reads is somewhat higher in the hotspot regions (Dataset S2). This suggests that both coding and non-coding regions of circular viral dsDNA are transcribed in both orientations to generate dsRNA precursors of viral siRNAs. Since genetic evidence revealed that the silencing-related, DNA-dependent RNA polymerases Pol IV and Pol V, or RNA-dependent RNA polymerases RDR1, RDR2 and RDR6 are not required for the biogenesis of CaMV- and CaLCuV-derived siRNAs [17], [18], these dsRNA precursors are likely generated by Pol II-mediated sense and antisense transcription. However, potential involvement of RDR3, RDR4 or RDR5 in viral siRNA biogenesis was not ruled out yet. In conclusion, similar to RNA viruses, the entire genomes of DNA viruses from the families *Caulimoviridae* and *Geminiviridae* are densely covered with non-redundant viral siRNA species and therefore can potentially be reconstructed as single contigs of the viral siRNAs.

**Figure 2:**
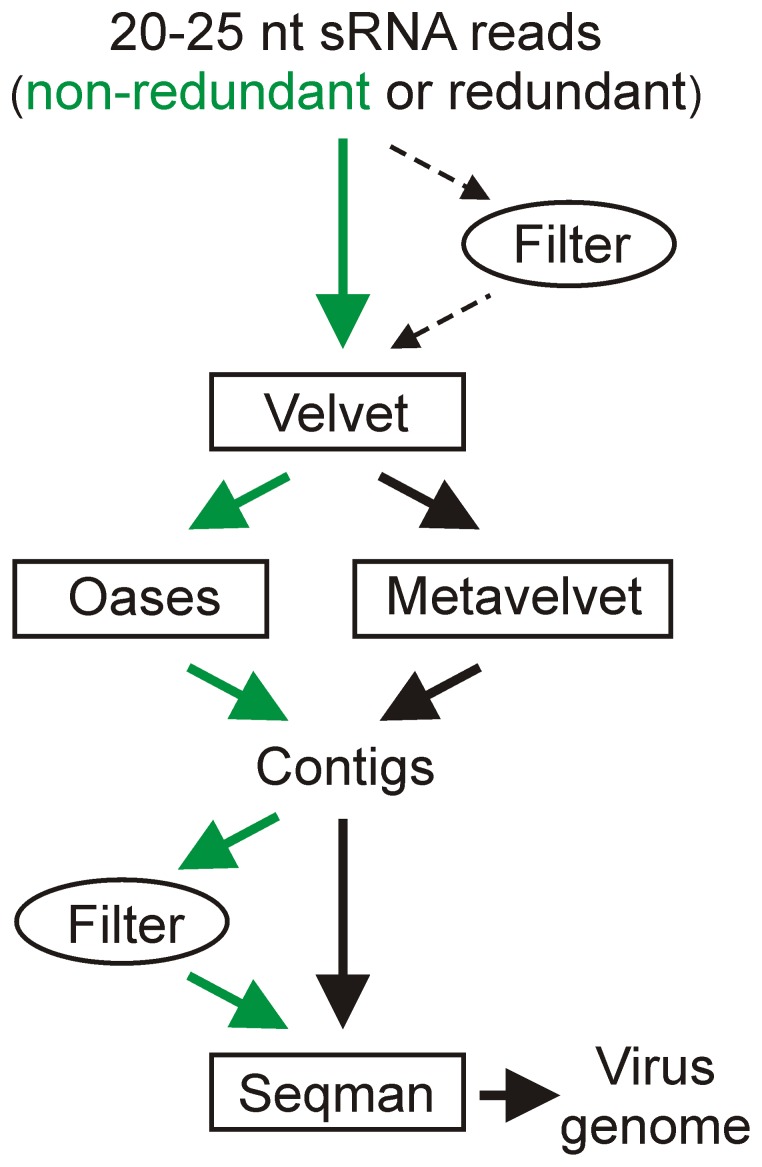
Bioinformatics algorithms for *de novo* reconstruction of viral/viroid genomes from siRNAs. The *de novo* assembler programs are boxed. Green arrows indicate the algorithm which in many cases generated the longest contigs.

To *de novo* assemble viral siRNAs, we tested different algorithms using Velvet followed by Oases or Metavelvet for assembling redundant or non-redundant reads and Seqman for merging the resulting contigs. In some cases, we also used mapping to the plant genome as a filtering step before Seqman to separate the viral siRNA contigs from the plant sRNA contigs (Figure 2). As a result, with both Oases and Metavelvet, the complete 8,031 nt genome of CaMV was reconstructed as a single terminally-redundant contig (Figure 1). The bipartite genome of CaLCuV was assembled as one terminally-redundant contig covering 2,512 nt DNA-B and two contigs covering 2,583 nt DNA-A (Figure 1). In the latter case, the filtering step was required. Because DNA-A and DNA-B of CaLCuV share a near identical common region of 195 nts (with 7 SNPs), during *de novo* assembly the DNA-A siRNA contig gets split in two contigs within this region. Generally, Oases generated longer contigs, while Metavelvet more precise contigs. Non-redundant reads assembled in longer contigs. SNPs and short indels that occurred in some of the contigs could be identified and corrected by SNP calling with redundant reads (see Materials and Methods for further details of the bioinformatics analysis). Thus, using Oases or Metavelvet followed by Seqman, the complete viral genomes were assembled *de novo* as single contigs of non-redundant siRNAs. This is unlike most of the above mentioned reports of virus or viroid reconstruction, in which multiple contigs of redundant siRNAs generated by Velvet were assembled using a reference genome as a scaffold. Furthermore, we found that the filtering step before Velvet, which was applied in some previous studies in efforts to remove host small RNAs interfering with assembly of viral siRNAs, often generates gaps in viral siRNA contigs. In contrast, the filtering step before Seqman used in our study enables reconstruction of complete genomes, especially in the case of DNA viruses. Moreover, we found that SNP calling with redundant siRNA reads can be applied to correct potential errors in *de novo* assembly algorithms.

**Figure 3:**
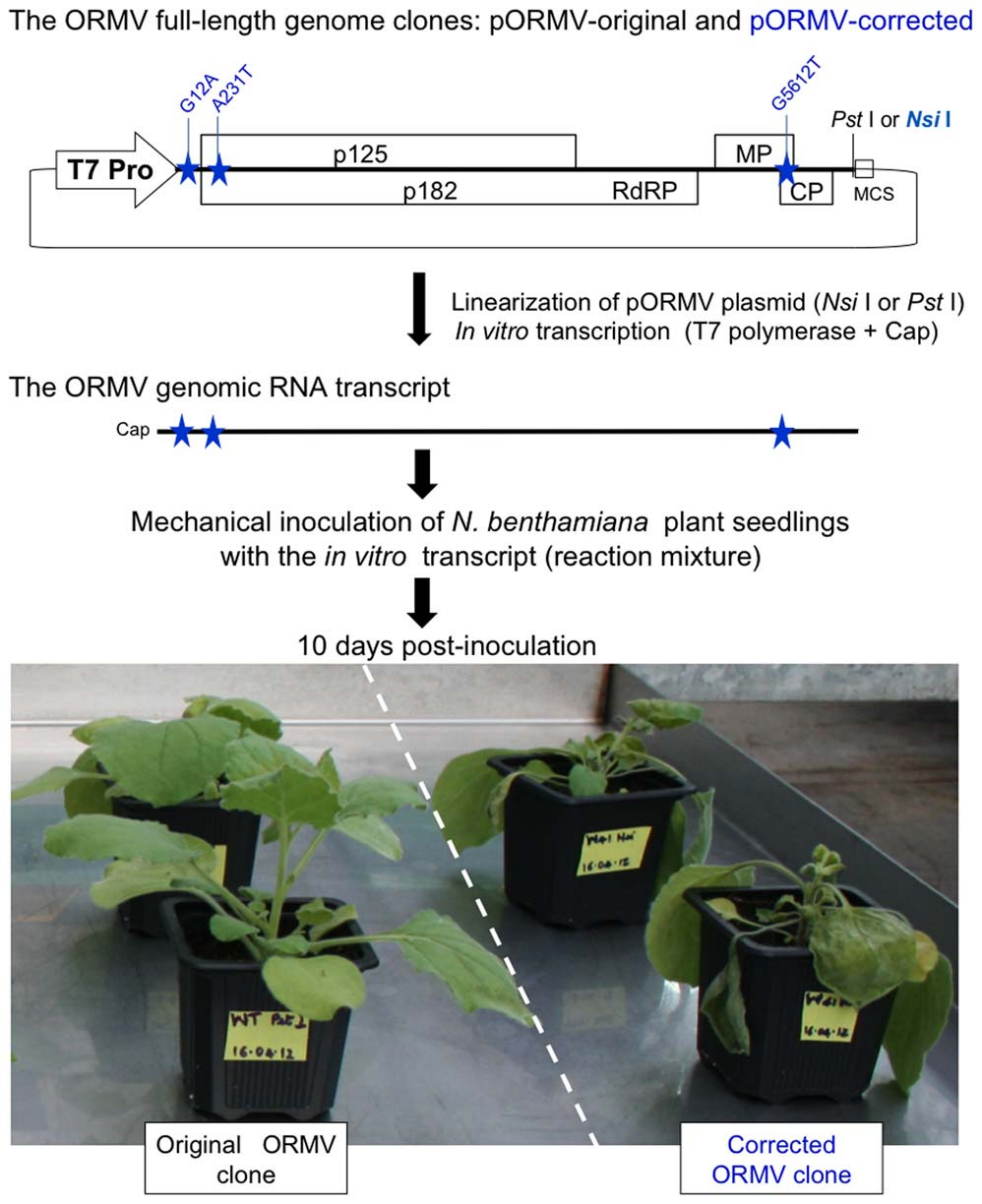
Test of the original and the corrected ORMV clones for infectivity. The plasmid containing the full-length ORMV genome sequence (original or corrected) behind the T7 promoter is depicted schematically: the restriction site *Pst* I or *Nsi* I, respectively, just downstream of the genome (located in multiple cloning site; MCS) was used for linearization of the plasmid, followed by run-off transcription by T7 polymerase in the presence of a cap analog. The resulting *in vitro* transcript (ORMV genomic RNA) was taken for mechanical inoculation of *N. benthamiana* plants. The picture shows the inoculated plants at 10 days post-inoculation.

To determine if such ‘siRNA omics’ (siRomics) approach is applicable for identification of a master genome in viral ‘quasispecies cloud’, we sequenced sRNAs from *Arabidopsis* infected with *Oilseed rape mosaic virus* (ORMV), the RNA virus for which an available cDNA clone was not infectious [20] because of potential cloning errors or because it represented a defective microvariant from the ORMV quasispecies. Using Oases followed by Seqman, the 6,303 nt ORMV genome was reconstructed *de novo* as a single contig from two independent sRNA libraries (Dataset S1*C* and Dataset S2). This reconstructed genome differed from the available cDNA sequence at three positions (G-to-A at position 12, A-to-T at position 231, and G-to-T at position 5612; Figure 1). SNP calling using the two sRNA libraries confirmed these mismatches in 96.5–97.7% (A12), 99.5–99.9% (T231) and 88.6–93.8% (T5612) viral redundant reads and highlighted the overall variation in the ORMV quasispecies (Dataset S3*A*). We corrected these mismatches (presumably cloning errors) in the cDNA clone and tested it for infectivity. Strikingly, the resulting clone was fully biologically active, causing the disease symptoms indistinguishable from those of the wild type ORMV sap (Figure 3). Thus, a common problem of virology, often taking years to overcome [21], was solved in one step. Our unpublished results suggest that G at position 12 in the original cDNA clone had a drastic impact on ORMV infectivity, possibly because it affects initiation of positive stand synthesis during the viral replication process; the nucleotide substitutions at the positions 231 and 5612 likely represent viable variants in the virus quasispecies (N.M.L., R.R., and M.M.P., in preparation).

Interestingly, SNP calling revealed that ORMV, CaMV and CaLCuV do not differ drastically in the frequency of SNPs or the average degree of deviation (in %) from the master genome nucleotides (Dataset S3*A-D*). This implies that distinct replication mechanisms of these viruses, involving the viral RDR (ORMV), the viral reverse transcriptase (CaMV), or the host DNA polymerase (CaLCuV), may have a comparable error rate. The error rate of the host DNA polymerase possessing proof-reading activity might be as high as the error rates of the viral RDR and reverse transcriptase lacking any proof-reading activity, because it replicates viral DNA via a rolling circle mechanism involving a viral Rep protein (recently reviewed by Pooggin [22]). Alternatively, comparable accumulation of the microvariants in all the three viruses may reflect their adaptation to the experimental host plant *Arabidopsis thaliana*. Note, that for identification of the SNPs listed in Dataset S3, we used a quite conservative cut-off, 10% non-redundant reads, to account for both an error of Illumina sequencing of sRNAs (0.1–0.5%) and sequence-specific biases that may lead to overrepresentation of certain sRNAs in redundant reads. Since host RDR activity may amplify CaLCuV siRNAs and thereby contribute to the observed deviations from the CaLCuV master genome, we compared the viral microvariant accumulation in CaLCuV-infected wild-type plants and *rdr1/2/6* triple mutant plants with diminished RDR activities [17]: no drastic difference was observed in the frequency of SNPs or the average degree of deviation from the master genome nucleotides (Dataset S3*C-D*). Our findings for CaLCuV are consistent with the observations that geminiviruses have high mutation frequency and evolve as fast as RNA viruses (see [23] and references therein).

**Figure 4:**
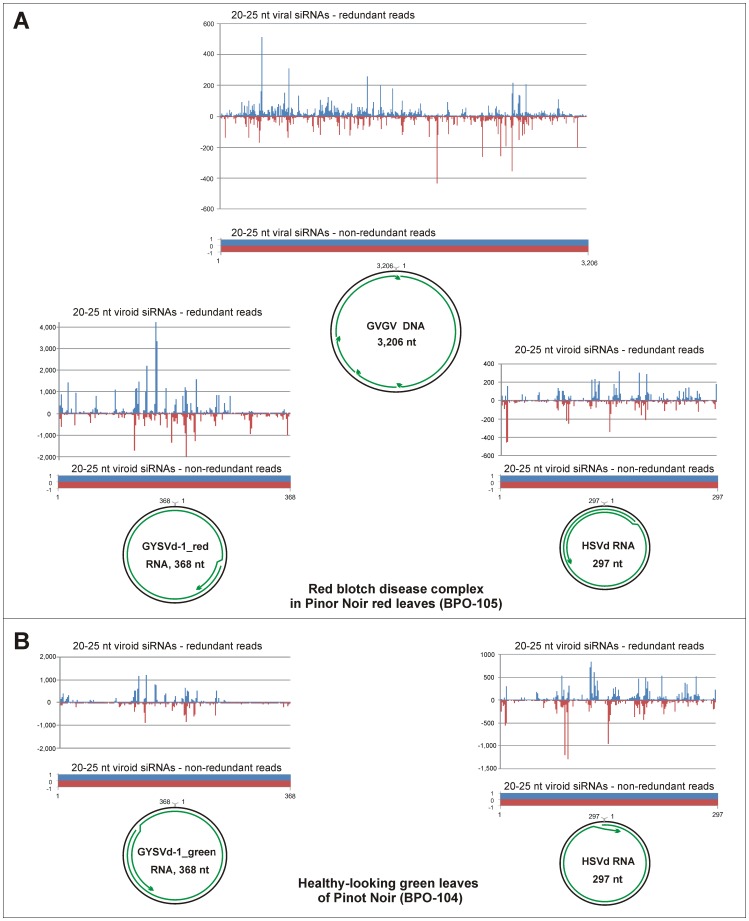
Maps of viral and viroid siRNAs and their contigs from red blotch disease-infected and healthy-looking leaves of grapevine. (**A**) Red blotch disease-infected leaves. (**B**) Healthy-looking green leaves. The graphs plot the number of 20–25 nt viral or viroid siRNA reads (redundant and non-redundant) at each nucleotide position of the genomes of the grapevine geminivirus (GVGV; also named GRBaV and GRLaV) and the viroids HSVd, GYSVd1_red and GYSVd1_green; Bars above the axis represent sense reads starting at respective positions; those below represent antisense reads ending at respective positions. The circular DNA genome of GVGV and the circular RNA genomes of HSVd, GYSVd1_red and GYSVd1_green are shown below the graphs, with the siRNA contigs covering the genomes depicted as green lines with arrowheads.

To evaluate the potential of siRomics for diagnostics of an unknown disease, we deep sequenced sRNAs from grapevines (*Vitis vinifera* cv. Pinot noir) grown at vineyards in Oregon, some of which exhibited severe leaf red blotch disease symptoms of unknown etiology, and from control plants with green, healthy-looking leaves. *De novo* reconstruction revealed that both infected and healthy-looking vines harbored *Grapevine yellow speckle viroid 1* (GYSVd-1) and *Hop stunt viroid* (HSVd), whose small circular RNA genomes were assembled as single terminally-redundant contigs and verified by redundant sRNA coverage (Figure 4; Dataset S1*D* and Dataset S2). Previously, these two viroids have been identified in grapevines in Oregon and elsewhere (see Materials and Methods). In addition, the disease-affected vines harbored a virus with a 3,206 nt circular genome reconstructed *de novo* from four contigs and validated by redundant sRNA coverage (Figure 4A; Dataset S1*D* and Dataset S2). Phylogenomic analysis showed that the virus is distantly related to circular DNA genomes of plant geminiviruses. The only closely related genome at the time of our analysis was that of a recently identified DNA virus from a declining Cabernet franc vineyard in New York State [24] (named ‘grapevine geminivirus’ (GVGV); the NCBI Genbank accession NC_017918). Despite their occurrence across the continent in distinct grapevine cultivars, the New York and Oregonian isolates differed by only 11 SNPs (Dataset S3*E*). Notably, all these 11 nucleotides in the Oregonian isolate are supported by at least 90% redundant reads in three independent red blotch leaf samples and therefore represent quite stable nucleotide positions in the master genome, unlike some of the other positions identified by SNP calling (Dataset S3*B*). We designed PCR primers specific to the virus (5′-TGCAAGTGGACATACGTTTA and 5′-GGGATCCCATCAATTGTTCT) and confirmed its presence in DNA samples from 12 of 16 symptomatic vines from the same vineyard, but not from any of 18 symptomless vines. Intriguingly, the most recent reports describe *Grapevine red blotch-associated virus* (GRBaV) and *Grapevine red leaf-associated virus* (GRLaV) that severely affect vineyards in States of California [25] and Washington [26], respectively. Both GRBaV and GRLaV sequences were found to be very similar to the geminivirus from New York [25], [26]. Thus, the virus that we identified in Oregon appears to be geographically wide-spread and associated with the emerging disease that threatens the high cash-value crop, grapevine. Whether this virus causes red blotch disease alone or in complex with the viroids identified in our study remains to be investigated. Interestingly, GRLaV was found to be associated with two RNA viruses and four viroids including HSVd and GYSVd-1 [26]. Thus, both or one of the latter two viroids may contribute to the disease.

Unlike most RNA viruses that generate predominantly 21-nt siRNAs (e.g. ORMV [4]; Dataset S1*C*), DNA viruses that are transcribed in the nucleus spawn 21-, 22- and 24-nt siRNAs (e.g. CaMV and CaLCuV; Dataset S1*A* and Dataset S1*B*), which are processed by distinct Dicer-like (DCL) enzymes from long dsRNA precursors [4], [17], [18]. We found that the grapevine geminivirus also induces formation of predominantly 21-, 22- and 24-nt siRNAs (Dataset S1*D*), thus resembling other geminiviruses. Furthermore, both coding and non-coding regions of this virus are covered with viral siRNA species in both orientations without gaps (Figure 4; Dataset S2). This suggests that bi-directional readthrough transcription of circular viral DNA may generate dsRNA precursors covering the entire virus genome, like it was proposed in the case of CaLCuV [17]. Analysis of 5′-nucleotides of the grapevine geminivirus-derived siRNAs and the host sRNAs (Dataset S1*D*) revealed similar biases to 5′U in 21-nt sRNAs and 5′A in 24-nt sRNAs. This indicates that, similar to plant miRNAs and siRNAs [27], [28], viral siRNAs are also sorted by Argonaute (AGO) proteins based on 5′-nucleotide identity to form silencing complexes. Bioinformatics analysis of siRNAs derived from the grapevine viroids HSVd and GYSVd-1 (Dataset S1*C*) revealed that these nucleus-localized viroids produce predominantly 21-, 22- and 24-nt siRNAs reminiscent to those produced by DNA viruses transcribed in the nucleus. In further similarity to DNA viruses, siRNA species of each size-class and polarity densely cover the entire circular RNA genomes of these viroids (Dataset S2; Figure 4). This implies that viroid siRNAs are processed from dsRNA intermediates of Pol II-mediated replication of circular single-stranded viroid RNA [29]. The hotspots of viroid/virus siRNA production that map to similar locations for each siRNA size-class and polarity (Dataset S2) may result from preferential internal processing of the dsRNA precursors by distinct DCLs and/or stabilization of siRNAs with certain nucleotide compositions by AGO proteins.

In summary, using deep siRNA sequencing and bioinformatics, dubbed siRomics, we developed a pipeline for nucleotide sequencing of the entire genomes of DNA and RNA viruses or viroids and for identification of the consensus master genome and its microvariants in viral or viroid quasispecies. Moreover, we demonstrated utility of siRomics for the universal diagnostics of known and emerging viral diseases, as well as for rapid generation of the biologically-active clones of problematic viruses. In addition to highlighting the potential of siRomics for applied research, our findings contribute to further understanding the siRNA silencing mechanisms targeting DNA viruses as well as RNA viruses and viroids.

## Materials and Methods

### Plants and viruses

Growth conditions and virus infections of *Arabidopsis thaliana* Col-0 plants were described in detail previously [4]. Briefly, seedlings were infected either by biolistic inoculation with DNA clones of *Cauliflower mosaic virus* (CaMV; the NCBI Genbank accession V00140) and *Cabbage leaf curl virus* (CaLCuV; U65529.2 for DNA-A and U65530.2 for DNA-B) or by mechanical inoculation with sap from *Oilseed rape mosaic virus* (ORMV)-infected *Nicotiana benthamiana*. A previously constructed plasmid containing ORMV cDNA downstream of the T7 promoter (kindly provided by Dr. Fernando Ponz) was modified using synthetic DNA fragments and suitable restriction sites to correct the cloning errors and obtain the reconstructed wild type ORMV genome clone (deposited to the Genbank as KF137561). The resulting and the original clones were linearized downstream of the ORMV sequence and used as templates for *in vitro* transcription reactions (MEGAscript T7 kit, Ambion) to produce a capped viral genomic RNA. The reaction mixtures were used for mechanical inoculation. Symptom development at day 10 post-inoculation is shown in Figure 3.

Samples of the red leaves displaying leafroll-like disease symptoms (named ‘red blotch’ disease) and healthy-looking green leaves of grapevine cv. Pinot noir plants were collected in a privately owned vineyard near Newberg, Oregon, USA in summer, 2011. The samples were scion clone 777 grafted onto rootstock 44–53 and collected with permission of the owner.

### Deep sequencing and bioinformatics analysis of viral/viroid siRNAs

Total RNA from infected and control tissue samples was extracted with Trizol and used for Illumina sequencing of 19–30 nt RNAs as described for CaLCuV by Aregger *et al*. [17]. The resulting small RNA (sRNA) libraries (detailed in Dataset S1) were taken for bioinformatics analysis and for *de novo* reconstruction of the viral genomes using the algorithms summarized in Figure 2. The results of bioinformatics analysis of the viral and host sRNA populations are summarized in Datasets S1, S2 and S3. To reconstruct viral and viroid genomes, the non-redundant or redundant sRNA reads ranging from 20 to 25 nts were assembled into contigs using Velvet 1.2.07 (www.ebi.ac.uk/~zerbino/velvet) [30] followed by Oases 0.2.08 (www.ebi.ac.uk/~zerbino/oases) [31] or Metavelvet 1.2.01 (metavelvet.dna.bio.keio.ac.jp) [32]. Number and size of the resulting contigs varied depending on the choice of Velvet *k*-mer values (13 through 23). 100% coverage of a virus genome could be achieved either with single *k*-mers or certain combinations thereof, as exemplified for CaMV, CaLCuV and ORMV in Dataset S4. SNP calling and correction of errors in viral contigs/genomes was done using Integrative Genomics Viewer (IGV; www.broadinstitute.org/igv) [33]. Oases and Metavelvet contigs obtained with all *k*-mer values or their selected combinations were merged using the Seqman module of Lasergene DNASTAR 8.1.2 Core Suite (DNAStar, Madison, WI). If required, the filtering step before Seqman (or Velvet) was done by mapping contigs (or sRNA sets) to the *Arabidopsis thaliana* genome (TAIR9) or *Vitis vinifera* genome (PRJNA33471) using Burrows-Wheeler Aligner (BWA) 0.5.9 [34]. The *de novo* reconstructed viral genomes were scanned for SNPs and indels using IGV with redundant reads. Finally, single-base resolution maps of viral sRNAs on the virus genomes were created using BWA and a sRNA map tool MISIS (www.fasteris.com/apps; [35]). Reads mapping to several positions on the reference sequence were attributed at random to one of them. To account for a circular virus/viroid genome the first 25 bases of the genome sequence were added to its 3′-end. For each reference genome and each sRNA size (20 to 25 nt), MISIS counted total number of reads, reads in forward and reverse orientation (Dataset S1) and thus generated single-base resolution maps (Dataset S2), where for each position starting from the 5′ end of the reference genome, the number of matches starting at this position in forward (first base of the read) and reverse (last base of the read) orientation for each read length is given. The reads mapped to the last 25 bases of the extended genome sequence were added to the reads mapped to the first 25 bases. MISIS generated two types of counts tables, one with zero mismatches and another with up to two mismatches. Comparison of the two tables was informative for identification and correction of potential mismatches between a reference sequence and the master genome sequence as well as for initial identification of SNPs and short indels in viral quasispecies (see Dataset S2, for all the viruses and viroids analyzed in this study). The positions of SNPs and the degree of deviation (in %) from the master genome nucleotide at each position in viral and viroid quasispecies were identified by IGV analysis of redundant and non-redundant sRNAs mapped to the reference genome with up to 2 mismatches. For identification of the SNPs listed in Dataset S3, we set an arbitrary cutoff value of 10% non-redundant reads: in other words, ten or more percent of the reads support the deviation from the master genome nucleotide for each SNP.

The analysis of siRNAs and complete genome contigs revealed that the infectious DNA-B clone of CaLCuV differs from its reference sequence U65530.2 by a single nucleotide deletion at the last position of the reference (making the genome 1 nt shorter), the infectious clone of CaMV differs from its reference sequence V00140 by two substitutions (C6175A and T6281C), while the original non-infectious ORMV clone differs from its reference sequence (NC_004422; named *Youcai mosaic virus*) at several positions. These apparent sequencing errors were confirmed by re-sequencing of the three clones. The original ORMV clone (confirmed by re-sequencing) differs at the three positions (Dataset S3*A*) from the reconstructed wild-type ORMV genome (KF137561) described in this study. *Hop stunt viroid* (HSVd; deposited to the Genbank as KF137565), which we reconstructed from each of the two green and three red leaf samples of grapevine cv. Pinot noir (Dataset S1*D*), is 100% identical to the sequences of other HSVd isolates, e.g. from grapevine cultivars Lumunage and Thompson Seedless in China (DQ371455, DQ371459) and a citrus tree in Tunisia (GU825977). In addition, the green leaves contained a variant of this viroid with the two SNPs supported by ca. 60% reads (Dataset S3*H*). *Grapevine yellow speckle viroid 1* (GYSVd-1) reconstructed from the two green leaf samples (GYSVd1_green; deposited to the Genbank as KF137564) is most closely related to the GYSVd-1 isolate from Germany (X87911; two SNPs), while GYSVd-1 reconstructed from the three red leaf samples (GYSVd1_red; deposited to the Genbank as KF137563) to the GYSVd-1 isolate from Japan (AB028466; two SNPs). The genome sequence of grapevine geminivirus (GVGV) reconstructed from the red leaves was deposited to the Genbank as KF137562.

## Acknowledgments

We thank Thomas Boller for supporting the research of M.M.P. group at the Botanical Institute, and Fernando Ponz and Manfred Heinlein for providing ORMV materials.

## Supporting Information

Dataset S1 Counts of viral and endogenous sRNAs in the sRNA deep-sequencing libraries from mock-inoculated and CaMV-infected *Arabidopsis* (Table S1A), CaLCuV-infected *Arabidopsis* (Table S1B), ORMV-infected *Arabidopsis* (Table S1C), and healthy-looking green and red blotch disease-infected leaves of grapevine cv. Pinot noir plants (Table S1D).
(XLSX)

Dataset S2 MISIS-generated, single-base resolution maps of 20–25 nt viral siRNAs from CaMV (BPO-20, BPO-22)-, CaLCuV (BPO-57)- and ORMV (BPO-38, BPO-44)- infected *Arabidopsis* plants and of 20–25 nt viral (GVGV) and viroid (HSVd, GYSVd1_red, GYSVd1_green) siRNAs from red blotch disease-infected red leaves (BPO-105) and healthy-looking green leaves (BPO-104) of grapevine cv. Pinot noir plants. The numbering of nucleotide positions are according to the NCBI Genbank reverence sequences of CaMV (V00140; note that two corrections C6175A and T6281C were introduced in this sequence based on the sRNA and DNA sequencing), CaLCV DNA-A (U65529.2), CaLCuV DNA-B (U65530.2; the last nucleotide of this reference sequence, position 2513, was deleted based on the sRNA and DNA sequencing), ORMV (KF137561), GVGV (KF137562), HSVd (KF137565), GYSVd1_green (KF137564) and GYSVd1_red (KF137563). Note that the positions of 5′-terminal nucleotide of sense sRNAs and 3′-terminal nucleotide of antisense sRNAs along the reference sequence are given, and the read counts are given for each sRNA of 20-, 21-, 22-, 23-, 24- and 25-nt classes mapped to the forward strand (X20, X21, X22, X23, X24, X25) and the reverse strand (X20_rev, X21_rev, X22_rev, X23_rev, X24_rev, X25_rev) with zero mismatches, along with the total counts of 20–25 nt sRNAs mapped on the forward (total_forward) and reverse (total_reverse) strands and on both strands (total). The last column contains the total number of 20–25 nt sRNA mapped to the reference sequence with up to two mismatches.
(XLSX)

Dataset S3 SNPs in viral and viroid quasispecies. **Table S3A**: SNPs at positions of the mismatches between the reconstructed wild-type ORMV genome and the original ORMV genome clone as well as SNPs in the wild type ORMV viral quasispecies; **Table S3B**: SNPs in CaMV; **Table S3C**: SNPs in CaLCuV DNA-A; **Table S3D**: SNPs in CaLCuV DNA-B; **Table S3E**: SNPs at the positions of the mismatches between the GVGV-Oregon and the GVGV-New York genomes as well as other SNPs in the GVGV-Oregon quasispecies; **Table S3F**: SNPs in the GYSVd-1 (red) and green) quasispecies; **Table S3G**: SNPs in the GYSVd-1 (green) quasispecies; **Table S3H**: SNPs in the HSVd quasispecies.
(XLSX)

Dataset S4 Analysis of the contigs generated by Velvet and Oases using non-redundant 20–25 nt sRNA libraries from CaMV (BPO-20 and BPO-21)-, CaLCuV (BPO-57)- and ORMV (BPO38 and BPO44)-infected *Arabidopsis*. Coverage (in %) of the viral genome sequences with the siRNA contigs is calculated for single k-mer values and combination thereof.
(XLSX)
